# Reversible Brain Sagging Dementia: A Two Case Report

**DOI:** 10.1002/alz70857_104421

**Published:** 2025-12-25

**Authors:** Veronica Perez, Marcelo Jobet, Juan Pablo Cruz, Patricio Mellado, Rodrigo A Santibanez

**Affiliations:** ^1^ Neurology Department, Faculty of Medicine, Pontificia Universidad Católica de Chile, Santiago, Chile; ^2^ Memory Clinic, Neurology Service, Complejo Asistencial Doctor Sótero del Río, Santiago, Chile; ^3^ Radiology Department, Faculty of Medicine, Pontificia Universidad Católica de Chile, Santiago, Chile

## Abstract

**Background:**

Brain sagging dementia (BSD) is a rare clinical syndrome related to spontaneous intracranial hypotension (SIH) that results in cognitive changes resembling behavioral variant frontotemporal dementia (bvFTD). We present two cases of BSD, presented in our institution in Chile.

**Method:**

The first case is a 54‐year‐old male with personality changes, disinhibition, forgetfulness, and headache, progressive over three months. Magnetic resonance imaging of the brain (MRI) revealed signs of spontaneous intracranial hypotension (SIH). MRI of the whole spine revealed a T1‐T8 paraspinal fluid collection. He received a blind blood patch (BBP) but persisted symptomatic. A new MRI revealed SIH progression, and computed tomography myelography (CT‐M) revealed a T11‐T12 cerebrospinal fluid (CSF) leak. He received a targeted blood patch (TBP), and the follow‐up MRI one month later revealed a complete resolution of SIH´s signs. Our second case is a 62‐year‐old male with personality changes, disinhibition, hyperorality, repetitive movements, forgetfulness, and mild headache, progressive over two years. Brain MRI revealed signs of SIH, and a fluorodeoxyglucose positron emission tomography scan of the brain (PET‐FDG) showed hypometabolism of the temporal poles and orbitofrontal cortex. MRI of the spine showed no abnormality. He received a BBP but persisted symptomatic; therefore, a CT‐M was performed, revealing an L4‐L5 CSF leakage, and a TBP was performed.

**Result:**

From the clinical standpoint, in the first case, the behavioral and cognitive symptoms completely resolved after the TBP. In the second case, following TBP, the headache fully resolved. However, behavioral and cognitive symptoms, while significantly improved, did not completely resolve.

**Conclusion:**

Although rare, BSD should be considered as a differential diagnosis when a bvFTD is suspected. Headache should be considered a red flag for BSD; however, it can be absent. CT‐M appears superior to spine MRI for locating CSF leaks. In BSD, identifying the site of the CSF leak and performing a TBP seems to have better outcomes. Our results suggest that a shorter period of time between symptom onset and treatment results in better outcomes. In BSD, as a reversible cause of dementia, efforts must be made towards the correct diagnosis and timely treatment.

